# Detection of Merkel Cell Polyomavirus in Seborrheic Keratosis

**DOI:** 10.3389/fmicb.2017.02648

**Published:** 2018-01-09

**Authors:** Lisa M. Hillen, Dorit Rennspiess, Ernst-Jan Speel, Anke M. Haugg, Véronique Winnepenninckx, Axel zur Hausen

**Affiliations:** ^1^Department of Pathology and GROW-School for Oncology and Developmental Biology, Maastricht University Medical Center, Maastricht, Netherlands; ^2^Department of Gynaecology, University Hospital RWTH Aachen, Aachen, Germany

**Keywords:** seborrheic keratosis, Merkel cell polyomavirus, MCPyV, p16, fluorescence *in situ* hybridization, FISH, immunohistochemistry, IHC

## Abstract

Seborrheic keratosis (SK) is the most common benign cutaneous neoplasm. A subset shows increased p16 expression. Since SK shares several features with verruca vulgaris, e.g., increased p16 expression, human papillomaviruses (HPV) have been suggested as possible causal agents. However, a relevant association could not be established between HPV and SK. In the present study we aimed to investigate the presence of Merkel cell polyomavirus (MCPyV) in relation to p16 expression in SK. P16 expression was investigated using immunohistochemistry (IHC). Presence of MCPyV was assessed in 23 formalin-fixed paraffin-embedded tissue samples of SK by molecular techniques (i.e., PCR and FISH) and IHC. 16/23 SK showed strong to moderate p16 expression. 6/23 of SK were MCPyV positive by PCR which was confirmed by FISH. Of interest, two samples with strong FISH signals also showed MCPyV expression as tested by IHC. Samples with weaker signal intensity were negative in IHC. P16 expression was not associated with the presence of MCPyV. Concluding, the detection of MCPyV DNA by PCR and FISH in SK reflects the widespread prevalence of MCPyV in the skin. However, low detection rates exclude MCPyV as a major pathogenic factor in SK, most likely representing a coincidental infection. P16 IHC does not appear as useful adjunctive surrogate marker for the presence of MCPyV in SK.

## Introduction

Seborrheic keratosis (SK) is the most frequent benign human skin proliferation, with increasing incidence in the elderly. The preferential localization is on the chest, interscapular region, waistline, and forehead (Yeatman et al., 1997; Kyriakis et al., 2012). SK shares several clinical and histopathological features with verruca vulgaris and condyloma acuminatum including increased p16 expression which is a tumor-suppressor protein and cyclin-dependent kinase (cdk) inhibitor (Chazal et al., 2002; Hodges and Smoller, 2002; Nakamura and Nishioka, 2003; Genders et al., 2017). Assessment of p16 protein expression by immunohistochemistry (IHC) is frequently used as a surrogate marker for human papillomavirus (HPV) infection in genital and oropharyngeal cancers (Dehn et al., 2007; Shelton et al., 2017). HPV infection has been suggested as a possible causative agent in SK, however a higher prevalence of HPV was only detected in subgroups of genital SK in comparison to non-genital SK, thereby excluding HPV to play a major pathogenic role (Zhu et al., 1991, 1992; Gushi et al., 2003; Nakamura and Nishioka, 2003; Tardío et al., 2012). Limited data is available concerning a possible role of p16 expression in SK and association with Merkel cell polyomavirus (MCPyV; Andres et al., 2010; Mertz et al., 2010). Similar to HPV, MCPyV is composed of a circular double-stranded DNA genome, possesses an icosahedral capsid symmetry, a homologous LxCxE motive in the encoded Retinoblastoma (Rb) binding site (Felsani et al., 2006; Shuda et al., 2008), and has a similar genome size (approximately 5,600 bp for MCPyV and 8,000 bp for HPV). In high risk HPV infection the Rb protein is inactivated resulting in cell cycle progression. As a consequence p16 expression is upregulated. Approximately 80% of Merkel cell carcinomas (MCC), a rare highly aggressive neuroendocrine skin cancer, are associated with MCPyV infection (Feng et al., 2008; Kassem et al., 2008). Interestingly, MCC and SK share several risk factors, such as long-term sun exposure, older age, and immunosuppression. Based on these findings we investigated p16 IHC expression in SK and tested for the presence of MCPyV using IHC and molecular methods (FISH and PCR).

## Materials and methods

### Patients and tissues

Formalin-fixed and paraffin-embedded (FFPE) tissues of 23 skin specimens were included in this study. All respective samples had been excised for diagnostic and therapeutic reasons and were obtained from the Maastricht Pathology Tissue Collection (MPTC). All use of tissue and patient data was in agreement with the Dutch Code of Conduct for Observational Research with Personal Data (2004) and Tissue (Federatie van Medisch Wetenschappelijke Verenigingen, FMWV, https://www.federa.org/) with written informed consent from all subjects in accordance with the Declaration of Helsinki. The protocol was approved by the Maastricht Ethic Committee (MEC) group. Diagnoses were previously defined by histology in routine diagnostics and have been reviewed by 3 experienced dermatopathologists (VW, AzH, LMH). The patient group consisted of 9 men and 13 women (ages 34–79; mean 58.8). Further details of the clinicopathologic parameters are included in Table 1. Serial sections of the specimens were used for hematoxylin and eosin (H.E.) staining, IHC, fluorescence *in situ* hybridization (FISH) and DNA isolation. In addition 16 non-neoplastic skin specimens originating from patients undergoing plastic surgery were tested (ages 18–63 year; mean 41.4 year).

**Table 1:** Summary of clinicopathological data, immunohistochemical staining and molecular results.

**Lab ID**	**Age**	**G**	**Localization**	**P16 IHC**	**SCS**	**PCR MCPyV M1M2**	**PCR MCPyV VP1**	**FISH MCPyV**	**IHC MCPyV**
VS1.1	55	f	Breast	+	+	+	+	+	−
VS2.2	79	f	Back	++	+	−	−	NA	−
VS4.1	58	f	Head	++	+	−	−	−	−
VS5.1	49	f	Back	−	+	+	+	+	−
VS6.1	70	f	Back	−	+	−	−	−	−
VS8.1	52	f	Head	++	+	+	−	+	−
VS10.1	65	f	Breast	+	+	+	−	+	−
VS11.1	56	m	Head	+	+	+	−	++	+
VS12.1	34	f	Axilla	−	+	−	−	−	−
VS13.1	59	f	Head	++	+	+	−	++	+
VS14.1	58	m	NA	−	+	−	−	NA	NA
VS15.2	59	m	Back	+	+	−	−	NA	NA
VS16.2	68	f	Head	+	+	−	−	NA	NA
VS17.2	47	f	Head	++	+	−	−	NA	NA
VS19.2	49	f	Head	−	+	−	−	NA	NA
VS20.2	73	m	Back	−	+	−	−	NA	NA
VS25.2	60	f	Breast	+	+	−	−	NA	NA
VS30.2	73	m	Back	+	+	−	−	NA	NA
VS37.2	58	f	Breast	++	+	−	−	NA	NA
VS38.2	73	m	Back	++	+	−	−	NA	NA
VS53.2	58	m	Head	++	+	−	−	NA	NA
VS54.2	54	m	Back	−	+	−	−	NA	NA
VS56.2	45	m	Back	+	+	−	−	NA	NA
Total: 23 samples	Mean: 58.8 year, SD 10.6 year	f (*n* = 14, 60.9%) m (*n* = 9, 39.1%)		++ 8/23 (34.8%) + 8/23 (34.8%) − 7/23 (30.4%)		6/23 26.1%	2/23 8.7%	6/23 26.1%	2/23 8.7%

*f, female; FISH, fluorescence in situ hybridization using full length MCPyV as probe with ++, strong, +, moderate, and −, negative nuclear signal pattern; G, gender; p16 IHC, immunohistochemistry using p16 with ++, strong; +; moderate; −, negative staining pattern; Lab ID, laboratory identification; m, Male; M1M2 PCR, PCR amplifying the M1M2 PCR product (178 bp); NA, not applicable; SCS ladder, specimen control size ladder; SD, standard deviation; VP1, PCR amplifying the viral capsid protein 1 (VP1) gen (315 bp); y, years*.

### Immunohistochemistry (IHC)

**Figure 1:**
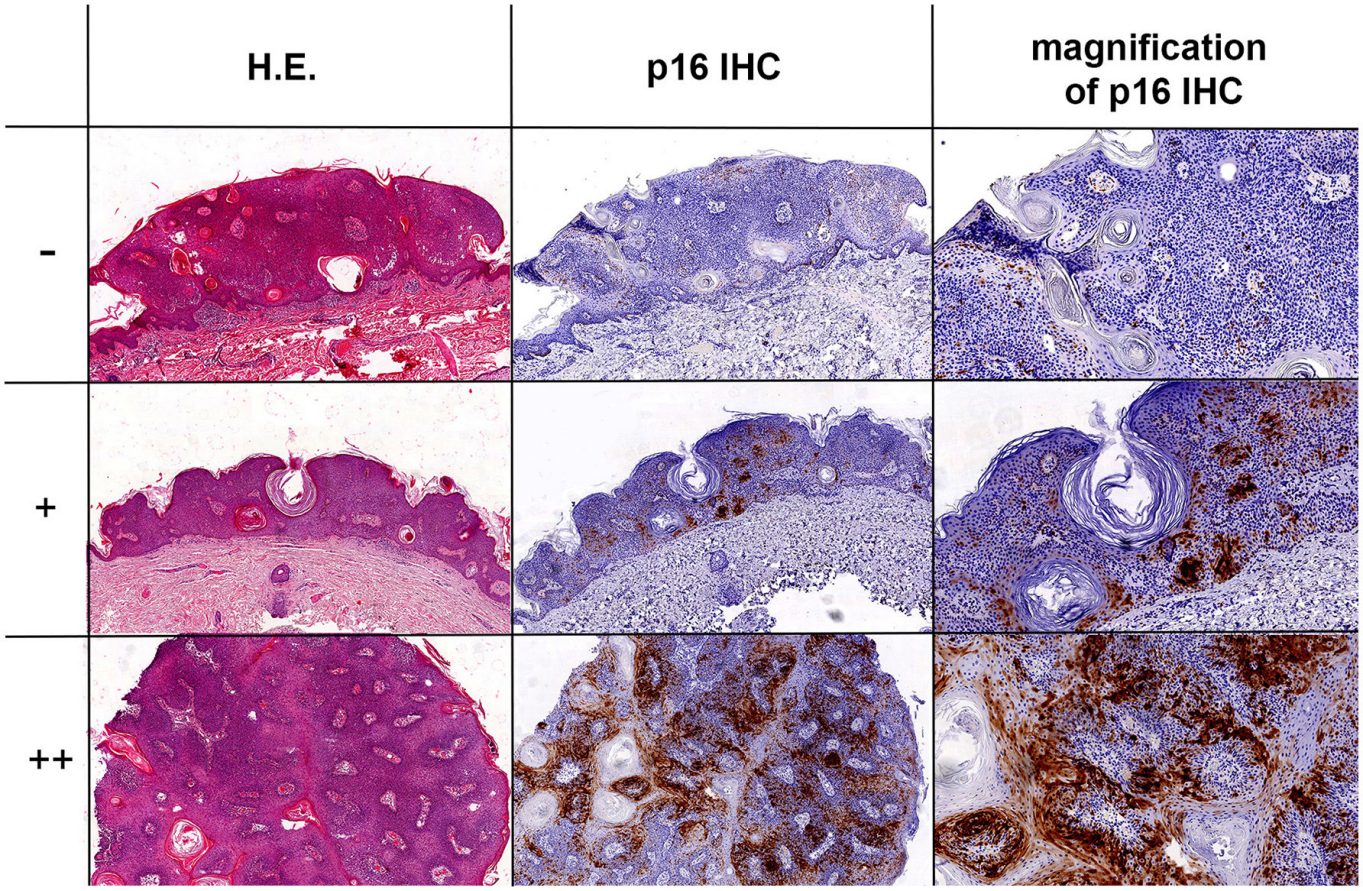
P16 IHC expression in SK. Three representative SK samples with H.E. staining and p16 IHC. The top images show a negative (–) score, center images a moderate (+) positive score, and bottom images a strong (++) positive score with nuclear and cytoplasmic staining for p16 in SK. The magnification of all pictures is 10x, with exception of the pictures in the very right row (top and center plot magnification: 20x; bottom plot magnification: 40x).

The following antibodies and dilutions were used in this study: anti-LT MCPyV (clone: CM2B4, dilution 1:50, Santa Cruz, Inc.), anti-p16 (clone: JCS, dilution 1:400, Santa Cruz, Inc.). Immunohistochemical stainings were conducted on a Dako Autostainer Link 48 using the EnVision FLEX Visualization Kit K8008 DAKO according to standard diagnostic routine protocols and manufacturers' instructions. P16 expression was correlated with the results obtained by MCPyV FISH and PCR data as described earlier (Hopman et al., 2005; Mertz et al., 2013; Haugg et al., 2014). P16 expression was evaluated by 4 experienced investigators (AzH, VW, DR, LH) with − as negative, + as moderate and ++ as strong positive score (Figure 1).

### DNA extraction

Five consecutive 10 μm thick sections were cut from each FFPE tissue. After deparaffinization, the tissues were lysed with proteinase K overnight (56°C) until complete tissue lysis, and DNA was extracted using a DNA Isolation QIAamp minikit (Qiagen, Hilden, Germany). Purified DNA was measured in a spectrophotometer (Nanodrop, 2000; Thermo Scientific). DNA quality and integrity were assessed by a specimen control size (SCS) ladder (Table 1) as described earlier (van Dongen et al., 2003). Inadequate samples were excluded from further study.

### Polymerase chain reaction (PCR)

PCR was performed with 150 ng of genomic DNA using the AmpliTaq Gold (Roche) DNA polymerase in a final volume of 50 μl. For MCPyV detection we used the VP1 and M1M2 primer sets and PCR conditions as published earlier (Feng et al., 2008; Kassem et al., 2008). Negative controls with water instead of patient samples were included in each amplification series.

### Sequence analysis

PCR products were submitted to automated nucleotide sequencing in an ABI 3130XL genetic analyser (ABI). DNA sequences were compared and analyzed with the reference sequences of the National Center for Biotechnology Information (NCBI) Entrez Nucleotide database gb EU375803.1 (MCC isolate 350) and gb EU375804.1 (MCC isolate 339) using the NCBI Blast program. Multiple sequence alignments were performed with Clustal W2 (EMBL-EBI-2015).

### MCPyV fluorescence *in situ* hybridization (FISH) probe

FISH was optimized and performed as described earlier (Hopman et al., 2005; Haugg et al., 2011, 2014). In brief, full length (5104 bp) MCPyV DNA was cloned into a StrataClone PCR Cloning Vector (pSC-A-amp/kan; Stratagene, Santa Clara, CA). The Plasmid DNA Purification Kit NucleoBond® PC 2000 (Macherey-Nagel, Dueren, Germany) was used to extract MCPyV plasmid DNA and sequenced using T7 and T3 primers. Labeling of the DNA was performed by standard nick translation with Biotin-Nick Translation Mix (Roche, Mannheim, Germany) containing biotin (Bio)-16-dUTPs. The final concentration of the labeled DNA was 2 ng/μl in 50% formamide, 20% dextran sulfate, 2x SSC pH 7.0, 50x excess carrier DNA from salmon sperm (Sigma Chemical, St. Louis, MO) and 50x tRNA from S. cerevisiae (Sigma Chemical, St. Louis, MO).

### Detection of MCPyV by FISH MCPyV

Deparaffinized 3 μm thick sections were pretreated for 20 min with 0.2 M HCl, incubated with 1 M NaSCN for 30 min at 80°C, washed in dH_2_O and 2x SCC and digested with 1 mg/ml pepsin (2,500–3,500 U/mg, Sigma Chemical, St. Louis, MO) in 0.14 M NaCl solution, pH2. The biotin labeled full length MCPyV DNA probe was added to the samples at a concentration of 5 ng/μl followed by denaturation of DNA (5 min, 80°C) and hybridization overnight (37°C, humid chamber, Thermobrite, Abbott, IL). Unbound MCPyV DNA probe was stringently washed away in 2x SSC, pH7 at 70°C for 2 min. Bound probe was detected by sequential incubation in a combination of fluorescein isothiocyanate (FITC) biotinylated avidin (AvFITC; 1:500; Vector, Brunswig Chemie, Amsterdam, The Netherlands) and biotin conjugated goat antiavidin (BioGaA; 1:100; Vector). Prior to incubation aspecific binding sites were blocked with Boehringer Blocking reagent. Cell nuclei were counterstained and coverslipped with 4,6-diamidino-2-phenylindole dihydrochloride (DAPI; 0.2 μg/ml, Vectashield, Vector Laboratories, CA). Samples were visualized using a DM 5000B fluorescence microscope (Leica, Wetzlar, Germany) coupled to an online digital camera (Leica DC 300 Fx) for independent evaluation of FISH signals by 3 investigators (AzH, DR, LH) according to criteria described earlier (Hafkamp et al., 2008; Haugg et al., 2014).

### Statistics

Data analysis was performed by SPSS statistical software (SPSS for windows, release 23.0; SPSS Inc., Chicago, IL, USA). Dichotomous variables were compared using the Pearson's chi-square test or Fisher's exact test as appropriate. The Spearman's rank correlation was used in nonparametric data to study the associations between different variables. A two-sided *P-value* less than 0.05 was considered statistically significant.

## Results

### P16 expression in SK

In total 16/23 (69.6%) of SK samples showed increased p16 expression in comparison to normal adjacent perilesional skin in all 16 samples. 8/23 (34.8%) showed strong (++) nuclear as well as cytoplasmatic p16 expression of the keratinocytes patchy scattered throughout the lesions. 8/23 (34.8%) showed a moderate (+) patchy p16 staining profile. 7/23 (30.4%) samples were negative for p16 expression (Figure 1 and Table 1).

### MCPyV detection in SK by PCR, FISH, and IHC

**Figure 2:**
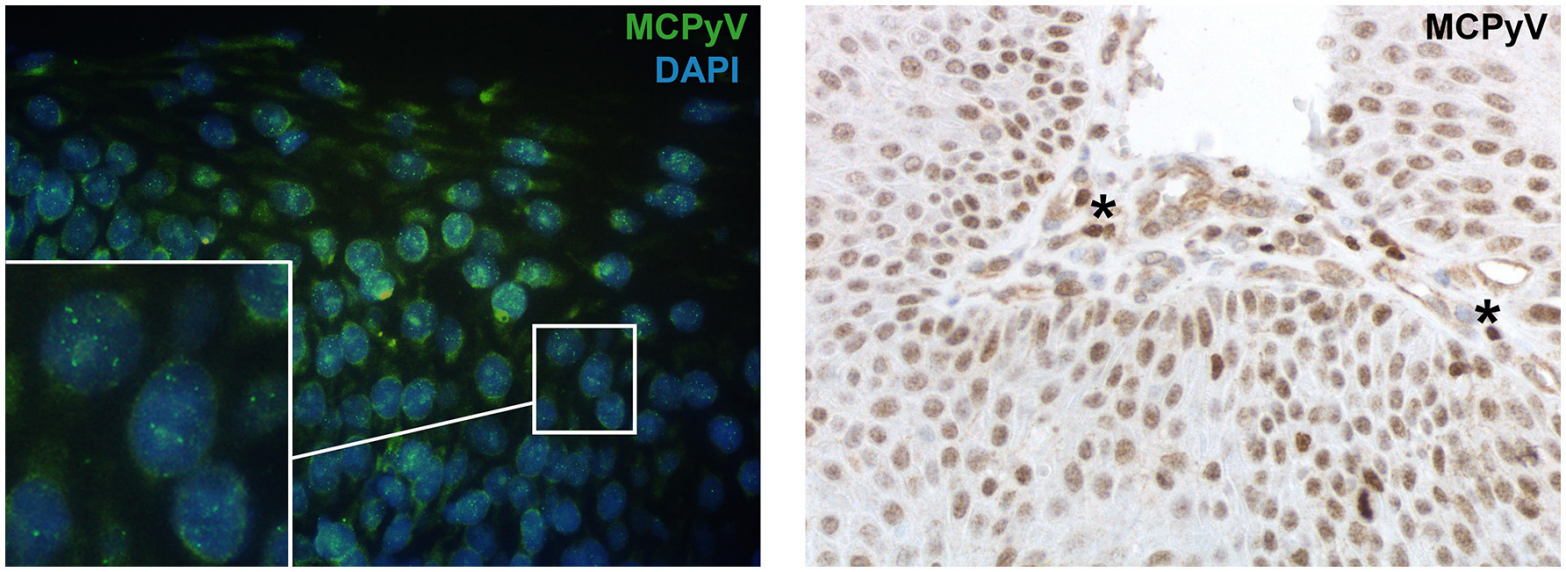
Presence of MCPyV detected by FISH and IHC in SK. **(A)** MCPyV DNA sequence nuclear hybridization signals visualized by FISH in the keratinocytes of the lesion (green); DAPI staining depicts the nuclei of the keratinocytes (blue) with a magnification of 63x. The white square shows a high magnification of nuclei to better illustrate the punctuate hybridization pattern. **(B)** IHC staining directed against MCPyV-LT antigen shows nuclear signals in the keratinocytes as well as in intracapillary blood cells, resembling lymphocytes (see ^*^) with a magnification of 40x.

PCR directed against diverse regions of the viral genome revealed that 6/23 (26.1%) of SK were MCPyV positive, including the viral M1M2 PCR product and in two cases the VP1 PCR product (Table 1). Sequence analyses of the PCR amplicons identified all PCR products as MCPyV DNA sequences. In the non-neoplastic skin specimens the presence of MCPyV PCR products was detected in 3/16 (18.8%) cases (data not shown). Results from PCR analyses could be confirmed by MCPyV FISH revealing nuclear hybridization with a punctual signal pattern in 6/23 (26.1%) of SK (Figure 2A and Table 1). In sample 11 and 13 there was a strong (++) nuclear signal pattern with FISH analyses. In sample 1, 5, 8, and 10 FISH signal intensity was moderate (+) and presence of MCPyV could be confirmed on PCR level, but was not detected by IHC. By IHC sample 11 and 13 (2/23, 8.7%) showed nuclear positivity for MCPyV large T (LT) antigen. The nuclear staining was seen in keratinocytes of SK and partly in surrounding intracapillary blood cells that resembled lymphocytes (Figure 2B). Molecular analyses with PCR and FISH were in good agreement (MCPyV M1M2 PCR vs. FISH with *p* = 0.03 and MCPyV VP1 PCR vs. FISH with *p* = 0.6). Importantly, specific nuclear hybridization signals of MCPyV were in several samples not only restricted to intralesional keratinocytes, but also seen in the adjacent preexistent skin. Thoroughly tested MCPyV-PCR negative cases subjected to FISH analyses and IHC staining remained negative for MCPyV.

### No correlation of P16 expression with MCPyV in SK

P16 expression was neither associated with presence of MCPyV on the DNA level (*p* = 0.69 for the M1M2 PCR product, *p* = 0.34 for the VP1 PCR product) nor on the translational level (*p* = 0.91). Furthermore there was no significant correlation of p16 expression with clinicopathological data (i.e., age, gender or localization of the lesions, Table 1).

## Discussion

In this study, we systematically evaluated p16 expression and the presence of MCPyV in SK and normal skin on the DNA and on the translational level by IHC. We were able to correlate molecular PCR results with the presence of viral DNA at a single cell level visualized by FISH analyses.

In our study 16/23 (69.6%) of SK showed a patchy moderate to strong p16 expression, and 7/23 (30.4%) of SK were negative for p16 in IHC. These findings are consistent with previous observations reporting 65% of SK with a patchy p16 expression profile and 35% of SK to be negative for p16 (Harvey et al., 2013). In another study 75% of benign keratotic lesions (including 11 SK and 5 verruca vulgaris) showed p16 staining including two SK lesions with intense p16 staining profile (Genders et al., 2017). Since the discovery of p16 in 1993 (Serrano et al., 1993), there has been an ongoing, controversial discussion concerning the meaning of increased p16 expression, which acts as a cyclin dependent kinase (CDK) inhibitor and specifically blocks CDK4 and CDK6. This blockade leads to decreased phosphorylation of Rb protein with subsequent arrest in the G1 phase of the cell cycle (Sharpless and DePinho, 1999). In general three major interpretational lines are followed in case of increased p16 expression in human epithelial cells: (i) p16 and its role as tumor progression marker (Nilsson et al., 2004; Conscience et al., 2006; Bagazgoitia et al., 2010; Harvey et al., 2013), (ii) p16 expression as virus surrogate marker in HPV associated lesions (Klaes et al., 2002; Nilsson et al., 2004; Horn et al., 2008; Schache et al., 2011), and (iii) p16 as a marker for cell senescence (Pavey et al., 1999, 2001; Nakamura and Nishioka, 2003; Vun et al., 2006; Liu et al., 2009; Donati et al., 2013; Shelton et al., 2017). In SK enhanced p16 expression has been mainly attributed to cell senescence and UV-exposure related photo-aging (Pavey et al., 1999, 2001; Nakamura and Nishioka, 2003; Liu et al., 2009; Donati et al., 2013). In the background of cutaneous lesions increased p16 expression has also been associated with malignant progression in actinic keratosis and Bowen's disease (Hodges and Smoller, 2002; Nilsson et al., 2004; Conscience et al., 2006; Bagazgoitia et al., 2010; Harvey et al., 2013). However, this interpretational approach seems not to account for increased p16 expression in SK, in which malignant transformation remains an extremely rare event (Vun et al., 2006; Rajabi et al., 2012; Conic et al., 2017). In the background of virus infection p16 IHC is a well-established surrogate marker for the diagnosis of HPV associated squamous cell neoplasms of the female genital tract and the oropharynx and the nasopharynx (Klaes et al., 2002; Horn et al., 2008; Schache et al., 2011; Vent et al., 2013). In line with this we hypothesized that the increased p16 in SK is possibly associated with a MCPyV infection in SK. However, in this study p16 expression did not correlate with presence of MCPyV.

To date there are only few data available on the prevalence of MCPyV in SK. Sample sizes of SK in other studies included at maximum 12 patients (Andres et al., 2010). Although the present study contains the largest sample number of SK (*n* = 23) and normal skin (*n* = 16) testing for the presence of MCPyV, the study number remains small and might represent a limitation. Designing this study, we aimed for a sample size of *n* = 20 for both groups. This number is statistically sufficient to screen if there is increased presence of MCPyV in relation to normal skin and if this correlates with p16 expression. A positive correlation was not identified such that the sample size has not been enlarged. Since normal skin by definition does not constitute an indication for a diagnostic procedure the number *n* = 20 could unfortunately not be sustained completely (*n* = 16).

Andres et al. reported an overall presence of MCPyV in 6% of sun-exposed non-MCC lesions. Interestingly both MCPyV positive non-MCC samples belonged to the SK-group (2 of 12 samples, 17%) while all lentigo maligna melanoma and basal cell carcinomas were negative for MCPyV (Andres et al., 2010). Concerning SK, this is in line with the findings in this study detecting the presence of MCPyV in 6 out of 23 samples (26.1%). Other reported data about presence of MCPyV in SK represent a case report of one patient with 3 SK samples. The patient was immunosuppressed with a MCPyV positive MCC. Mertz et al. found MCPyV sequences in all of this patients common warts (4/4), half of his carcinoma *in situ* lesions (3/6) and two SK (2/3; Mertz et al., 2010). Depending on the detection method, we found MCPyV in 8.7% of samples via IHC and in 26.1% by molecular analyses by PCR and FISH in SK. We could not demonstrate an increased prevalence of MCPyV in SK compared to the tested normal non-neoplastic skin samples (*n* = 16), thereby most likely excluding MCPyV from playing a major pathogenic role in SK.

In this study MCPyV PCR sequences were detected in 3/16 (18.8%) of normal skin samples. Data from the literature show high variability of MCPyV in normal skin with percentages between 0% (Garneski et al., 2009; Kassem et al., 2009; Mangana et al., 2010) and 78% (Loyo et al., 2010) with most data ranging from 17 to 24% (Wieland et al., 2009; Foulongne et al., 2010; Mertz et al., 2010). The differences in the reported prevalence between the studies may be caused by heterogeneity of the study population (immune status, age-, and gender distribution), as well as heterogeneity in performed methods for detection of viral load (i.e., primer selection, viral-DNA copy number, etc. Dworkin et al., 2009; Garneski et al., 2009; Wieland et al., 2012). In case of PCR as screening method, multiple factors may account for the variable MCPyV detection rates including tissue quality, quantity and fixation, DNA extraction method, PCR technique, choice of viral gene targets, and primer selection. A further limitation is the relatively low number of investigated samples from studies which have addressed normal, non-neoplastic, skin samples.

In line with other studies, the used MCPyV primer sets yielded partially heterogeneous results. Differences in detection frequency might be caused by changes due to the viral integration process (Andres et al., 2010; Kassem et al., 2010). In this study we were able to correlate molecular results from PCR to a single cell level and visualize MCPyV nucleic acids with FISH, thereby studying infected cells within the histological context. The two samples with strong punctual nuclear signal pattern in FISH analyses also showed IHC MCPyV expression on the translational level. PCR positive samples with weak signal intensity in FISH analyses were negative in IHC. This observation is in line with findings of several other studies which detected MCPyV on the IHC level to a lesser extent than by PCR (Katano et al., 2009; Shuda et al., 2009; Mertz et al., 2010; Ly et al., 2012). Discrepancies in MCPyV detection rates may be attributed to the enhanced sensitivity of PCR and FISH to detect low viral loads in contrast to IHC. Although unlikely, another reason for the lower detection rate with IHC might be a putative mutational loss of the antibody epitope. Indeed, the discrepancy in PCR and FISH compared to IHC is partly because PCR can target other viral components such as structural proteins VP1, VP2, and VP3, small T antigen and large T (LT) antigen, whereas the IHC derived from clone CM2B4 only targets the MCPyV LT antigen. Finally, although PCR effectively detects viral DNA, it does not distinguish incidental presence of virus from a causal infection. Comparatively, MCPyV FISH enables direct visualization of MCPyV DNA on a single cell level, while IHC allows the direct visualization of nuclear LT antigen expression only in the setting of relatively high viral load which may be more indicative of a causal infection (Ly et al., 2012; Haugg et al., 2014). Summarizing these findings indicate that MCPyV infection with low viral amplification rate (i.e., viral load) yields positive in FISH and PCR analyses but negative in IHC results.

Molecular analyses with FISH and observations in IHC revealed that in several samples MCPyV was not only restricted to intralesional keratinocytes and adjacent skin but also present in some intracapillary blood cells with a lymphocytic morphology. These observations indicate MCPyV to be ubiquitously present in diverse human tissue independently of malignant or benign histological status (Feng et al., 2008; Shuda et al., 2008).

The interpretation of the IHC stain for p16 may have been affected by pigmentation in the SKs. Therefore, substitution of the brownish dye for red as secondary antibody was used in strongly pigmented lesions to rule out the possibility of false-positive interpretations.

## Conclusion

The frequent detection of the MCPyV genome by PCR and FISH reflects ubiquitous spread of the virus. However, the low rate of MCPyV detection by IHC most likely excludes a major pathogenic association of MCPyV in SK development, similar to the lack of evidence for a role of MCPyV in other non-melanoma skin neoplasm. The sporadic detection most likely represents a coincidental infection with a ubiquitous virus (Loyo et al., 2010; Wieland et al., 2012; Mertz et al., 2013; Peretti et al., 2014). Our data indicate that p16 IHC is unlikely to be a helpful adjunctive biomarker in the detection of MCPyV infection.

## Author contributions

Designed the experiments: AzH, VW, and LH. Conceived and supervised the study: AzH, AMH, and VW. Performed the experiments: LH and DR. Contributed reagents, materials: E-JS and AMH. Data analyzed: LH, DR, AzH, and VW. Wrote the manuscript: LH and AzH. All authors read and approved the final manuscript.

### Conflict of interest statement

The authors declare that the research was conducted in the absence of any commercial or financial relationships that could be construed as a potential conflict of interest.
